# Electric Field-aided Selective Activation for Indium-Gallium-Zinc-Oxide Thin Film Transistors

**DOI:** 10.1038/srep35044

**Published:** 2016-10-11

**Authors:** Heesoo Lee, Ki Soo Chang, Young Jun Tak, Tae Soo Jung, Jeong Woo Park, Won-Gi Kim, Jusung Chung, Chan Bae Jeong, Hyun Jae Kim

**Affiliations:** 1 School of Electrical and Electronic Engineering, Yonsei University, 50 Yonsei-ro, Seodaemun-gu, Seoul 120-749, Republic of Korea; 2 Division of Scientific Instrumentation, Korea Basic Science Institute, 169-148 Gwahak-ro, Yuseong-gu, Daejeon 305-806, Republic of Korea

## Abstract

A new technique is proposed for the activation of low temperature amorphous InGaZnO thin film transistor (a-IGZO TFT) backplanes through application of a bias voltage and annealing at 130 °C simultaneously. In this ‘electrical activation’, the effects of annealing under bias are selectively focused in the channel region. Therefore, electrical activation can be an effective method for lower backplane processing temperatures from 280 °C to 130 °C. Devices fabricated with this method exhibit equivalent electrical properties to those of conventionally-fabricated samples. These results are analyzed electrically and thermodynamically using infrared microthermography. Various bias voltages are applied to the gate, source, and drain electrodes while samples are annealed at 130 °C for 1 hour. Without conventional high temperature annealing or electrical activation, current-voltage curves do not show transfer characteristics. However, electrically activated a-IGZO TFTs show superior electrical characteristics, comparable to the reference TFTs annealed at 280 °C for 1 hour. This effect is a result of the lower activation energy, and efficient transfer of electrical and thermal energy to a-IGZO TFTs. With this approach, superior low-temperature a-IGZO TFTs are fabricated successfully.

Transparent and flexible electronics are attracting interest for their potential application in next generation electronics including paper displays, wearable computers, and roll-up devices^1^. Amorphous InGaZnO thin film transistors (a-IGZO TFTs) have been extensively studied as their field-effect mobility (>10 cm^2^ V^−1^ s^−1^) is greater than a-Si equivalents (~1 cm^2^ V^−1^s^−1^), and the processing temperatures required are lower than for a-Si (~350 °C), and polycrystalline Si (~500 °C) TFTs^2^,^3^,^4^. Despite the stated advantages of a-IGZO TFTs, the low thermal resistance of flexible substrates can complicate processing. Sputtered a-IGZO TFTs have been reported to require an additional 300 °C annealing process after deposition of the a-IGZO active layers to obtain sufficient semiconductor properties and transfer characteristics^5^. Oxide films are deposited during the sputtering process, and defect sites are formed from high energy ion bombardment. These defects act as scattering centers or charge carrier traps, leading to degradation of the electrical characteristics of a-IGZO TFTs^6^,^7^,^8^,^9^. In, Ga, Zn, and O atoms require an activation energy (E_a_) sufficient to rearrange the metal oxide structure through high temperature annealing. Without activation through the annealing process, the sputtered a-IGZO layer does not have semiconducting characteristics. However, flexible plastic substrates with a low-glass transition temperature are not suitable for high temperature treatment^10^,^11^. The thermal budget is proportional to the annealing temperature, time, and area. For a 46 inch full-HD panel, only 0.66% of the panel area comprises the final active layer. The conventional activation process is wasteful as the entire panel is annealed, and over 99% of the active layer is etched after activation. Therefore, it is essential to develop an efficient, localized, low-temperature activation method to practically realize flexible electronics. However, previous researches on low-temperature activation such as UV annealing and high pressure annealing cannot overcome the same limitation, either. Here, we demonstrate a technique for the selective annealing of oxide films, using biased electrodes, to fabricate an a-IGZO backplane at 130 °C as shown in Fig. 1a. The electrical characteristics and chemical properties of electrically activated a-IGZO TFTs (EATs) were also investigated. Thermodynamic and electrical analysis of the a-IGZO channel will be used to explore the mechanisms of electrical activation.

## Results

### The electrical characteristics of thermally activated TFTs (TATs) and electrically activated TFTs (EATs)

Figure 2d shows the transfer characteristics of the samples annealed at several temperatures (80–280 °C) for 1 hour. The a-IGZO TFTs annealed at 280 °C (TATs, sample number 1) exhibited sufficient transfer characteristics, such as field-effect mobility (μ_FET_); it is known that annealing at temperatures around 300 °C is required to control the properties of AOS films and TFTs^12^.

Figure 2e shows the transfer characteristics of the non-activated (electrical) TFTs (V_G_ (−50) V_DS_ (0)_1, V_G_ (−50) V_DS_ (0)_2, V_G_ (−50) V_DS_ (−50), V_G_ (−50) V_DS_ (+50), V_G_ (−50) V_DS_ (+100), V_G_ (0) V_DS_ (0)_1, V_G_ (0) V_DS_ (0)_2, V_G_ (0) V_DS_ (+50), V_G_ (+50) V_DS_ (0)_1, V_G_ (+50) V_DS_ (0)_2, and V_G_ (+50) V_DS_ (−50)_0.5 h). Figure 2f shows the transfer characteristics of EATs (V_G_ (0) V_DS_ (−50), V_G_ (0) V_DS_ (+100), V_G_ (+50) V_DS_ (−50), V_G_ (+50) V_DS_ (+50), V_G_ (+50) V_DS_ (+100), and V_G_ (+50) V_DS_ (−50)_2 h). A non-activated (thermal) TFT (NAT) that was annealed at 130 °C for 1 hour without an applied bias in Fig. 2d and the EATs were annealed at the same temperature, but only the EATs demonstrated activated transfer characteristics.

The EATs passed a low current for negative V_G_, and a high current for positive V_G_, suggesting semiconductor characteristics. In contrast, the NAT demonstrated metallic characteristics for all tested gate biases.

Despite the low fabrication temperature, the μ_FET_, on/off ratio, V_on_, subthreshold swing (SS), and maximum trapped charge density (N_max_) of the EATs was similar to the TAT, as shown in Table 1. μ_FET_ was obtained from the saturation region of the I_DS_–V_DS_ curves (V_DS_ ≥ V_GS_−V_T_) using the equation


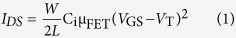


where C_i_ and V_T_ are the gate capacitance and threshold gate voltage respectively^13^. Figure S1 (Supplementary Information) shows a comparison of a-IGZO TFTs (TAT, V_G_ (−50) V_DS_ (−50), V_G_ (−50) V_DS_ (+50), V_G_ (−50) V_DS_ (+100), V_G_ (0) V_DS_ (−50), V_G_ (0) V_DS_ (+50), V_G_ (0) V_DS_ (+100), V_G_ (+50) V_DS_ (−50), V_G_ (+50) V_DS_ (+50), V_G_ (+50) V_DS_ (+100), and V_G_ (+50) V_DS_ (−50)_2 h) in terms of SS and μ_FET_, and the optimized conditions were determined by observation of improvements in these factors. The optimized sample conditions were V_G_ = 50 V, V_S_ = 0 V, and V_D_ = −50 V. The optimized EAT (sample number 14, V_G_ (+50) V_DS_ (−50)) characteristics were slightly better than the TAT: μ_FET_ increased from 9.90 to 10.66 cm^2^ V^−1^ s^−1^, and SS decreased from 0.42 to 0.36 shown in Table 1.

### Thermodynamic analysis of EATs

Infrared microthermography was used to investigate the heating between the source and drain electrodes. The measurement concept and experimental details are described in ref. 14. The maximum temperature change (∆T_max_) measured in the TFT channel of each sample is given Table 1. Figure 3a shows an image of V_G_ (+50) V_DS_ (−50), and Fig. 3b–d shows the TFT temperature distribution during the electrical activation process (V_G_ = 50 V). It can be seen that the annealing is focused in the predetermined channel regions. Self-heating occurs during the electrical activation process due to the high drain current, increasing the temperature of the channel region^15^,^16^,^17^.

Figure 3e shows ∆T_max_ of the a-IGZO channel when V_g_ = −50 V, 0 V, and 50 V. ∆T_max_ was seen to increase with |V_DS_| for each V_G_ condition. When V_DS_ = 0 V, no drain current flows, so Joule heating did not occur. Joule heating increased with the drain current that originates from V_G_ in accordance with Equation 1. A high positive V_G_ attracts more electron carriers, and generates heat. Negative V_G_ repels electrons, and results in the least Joule heating.

Joule heating is the process by which the flow of an electric current through a conductor produces heat. According to Joule’s first law, the amount of heat released is proportional to the square of the current such that


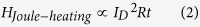


where R is the resistance of the material, and t is the duration of the current flow^18^. Based on the Equation (2), the amount of Joule heat increases as the material is low resistive (low oxygen concentration in oxide) and more current flows in the same voltage condition.

For a system with N particles, its thermal energy U_thermal energy_ is


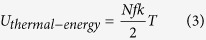


where *k* is the Boltzmann constant, *f* is the number of degrees of freedom, and T is absolute temperature^19^. During the electrical activation process, the temperature of the a-IGZO channel increased. If we assume that a system is a-IGZO TFT, its thermal energy can be changed as follow from the Equation 3.


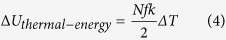


According to the first law of thermodynamics, the change in internal energy of a system (U) is equal to the heat (Q) added to the system minus the work (W) done by the system^20^,^21^,^22^,^23^.


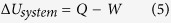


If we assume that no work is done by an a-IGZO channel, the variation in energy will be the heat energy added to the channel.


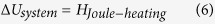


In our assumption, the increase in temperature of an a-IGZO channel is attributed to the heat generated by current flow


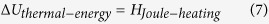


We can rewrite the Equation 7 by using Equations 2 and 4,


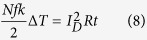


We assumed that N, f, k, R, and t are constant values, and T (temperature) changed by I_D_ (current) flowed.


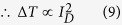


Therefore, it can be expected that the temperature change will be proportional to the square of the drain current, and the temporal behavior of the samples is in agreement with our expectations as shown in Fig. 3f, from which the following equation can be extrapolated.


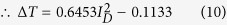


which states the relationship between the temperature change and drain current in the case of V_G_ = 50, V_S_ = 0, and V_D_ = −50 V. Also, this equation matches well with the data from V_G_ (+50) V_DS_ (−50).

### XPS and SE analysis of before (as-deposited) and after electrical activation

Figure 4a shows the valence band offsets (E_FV_ = E_F_ − E_V_) between the Fermi level (E_F_) and the valence band maximum (E_V_) using XPS data. Optical bandgap energies (E_g_) were derived from the SE data, as shown in Fig. 4b. The conduction band offsets (E_CF_ = E_C_ − E_F_) between the Fermi energy level (E_F_) and the conduction band minimum (E_C_) were calculated using the valence band offsets and bandgap energies. Figure 4c shows the band alignments measured before (as-deposited) and after the electrical activation process, and the E_CF_ results are given in Table S2 (Supplementary Information). The results indicate that the electrical characteristics of a-IGZO films vary with the carrier concentration. According to semiconductor physics theory, the electron concentration (n) can be described by the following equation:


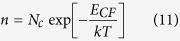


where N_c_ is the effective density of states at the conduction band edge. This equation shows that n increases exponentially as E_CF_ decreases. E_F_ before electrical activation is located near E_C_, and therefore pre-activation EATs with relatively small E_CF_ values have metallic properties due to the excessive n of the a-IGZO active layer. The E_F_ of post-activation EATs is separated from E_C_, suggesting that the E_CF_ of the EAT is increased after the electrical activation process, resulting in semiconductor characteristics with appropriate n in the a-IGZO active layer.

Figure 5a,b shows the O 1s peaks from XPS analysis for a-IGZO TFTs before (as-deposited) and after electrical activation, respectively. XPS data are calibrated using the C 1s peak centered at 284.8 eV, and the O 1s peaks were deconvolved into two peaks. The lower binding energy peak represents a metal oxide lattice without oxygen vacancy, centered at 530 ± 0.1 eV. The higher binding energy peak is related to non-lattice oxygen, and is centered at 531.9 ± 0.3 Ev^24^. The peak is the remaining peak sum after subtraction of the lattice oxygen peak from the total, and includes the peaks of oxygen vacancy and hydroxyl groups. In comparison with pre-activated EATs, EATs have more lattice oxygen (increased from 67.7% to 84.2%) and less non-lattice oxygen (decreased from 32.3% to 15.8%). This result of increased lattice oxygen indicates that electrical activation decomposes the weak metal oxide bonds, and rearranges atoms to form a chemical atomic network. Therefore, XPS and SE results demonstrate that electrical activation of a-IGZO TFT is effectively achieved^24^,^25^,^26^.

## Discussion

Based on results of electrical, thermodynamic and chemical analysis, we have compared and analyzed electrically activated samples in various voltage bias conditions (V_G_, V_S_, and V_D_) so that we can suggest mechanisms of electrical activation. Thermal energy sufficient for channel activation should be generated and selectively transferred to the channel area. Joule heating is caused by the motion of particles that form the current (typically electrons) and the ions that form the body of the conductor. Charged particles are accelerated by an electric field, and the increase in the kinetic vibration energy of colliding ions is transferred from the power supply (Fig. 6a).

The polarity of V_G_, and the electrical potential between the three electrodes must be considered. The polarity of V_G_ produces a Coulombic force. As V_G_ is made more positive, more carriers accumulate in the channel, causing a temperature increase (Fig. 6b). Therefore, the optimized V_G_ condition is at 50 V. For example, V_G_ (+50) V_DS_ (−50) experienced a temperature increase greater than that of V_G_ (−50) V_DS_ (−50), resulting from V_G_ = 50 V, although both devices had the same absolute voltages of V_G_, V_S_, and V_D_.

During the electrical activation process, the device temperature reached 58 °C, depending on various bias conditions. However, similar temperature increases occur when the signs and values of the applied voltages differed, as is clearly shown in Table S1 for V_G_ (0) V_DS_ (−50) and V_G_ (+50) V_DS_ (+50). Therefore, the potential differences |V_DS_|, V_GS_, and V_GD_ are also important. As discussed previously, |V_DS_| is required to be greater than 50 V (Fig. 6c). In addition, a potential difference of at least 50 V is needed between V_GS_ or V_DS_. For example, V_G_ (0) V_DS_ (−50) and V_G_ (+50) V_DS_ (+50) could be electrically activated as they had the same |V_DS_| of 50 V. V_GD_ of V_G_ (0) V_DS_ (−50) was 50 V, and V_GS_ of V_G_ (+50) V_DS_ (+50) was 50 V. Other samples could be activated if they had potential differences of more than 50 V including |V_DS_|.

In addition to an increase in substrate temperature, E_a_ is reduced by applying a bias during electrical activation. The activation temperature was decreased from 280 °C to 130 °C, a difference of 150 °C. However, the maximum temperature increase from Joule heating is 54 °C, suggesting that electrical activation may not be achieved by Joule heating alone. The a-IGZO layer comprises numerous electrons, protons, and ions of In, Ga, Zn, and O. Figure 7a shows how the charged particles experience attractive or repulsive forces, depending on the applied electric field. Therefore, external force is applied to the a-IGZO layer. As more external energy is added to the a-IGZO system, it transitions from a low-energy state to a metastable state^27^. This increase in the free energy of an a-IGZO system results in a decreased E_a_ (E_a_’ in Fig. 7b). The electric field is analogous to a chemical catalyst that lowers E_a_. However, a catalyst does not change the free energies of the original reactant or products; rather, the reactant energy and the product energy are maintained while E_a_ is altered. In contrast, electrical activation changes the free energy of the reactant, which is an a-IGZO film before activation. The activation of the chemical reaction by an applied electric field is controlled by the bias voltage. The electrical energy compensates for the insufficient thermal energy at low temperatures, so that the annealing temperature can be decreased without degradation of the electrical characteristics.

**Figure 1:**
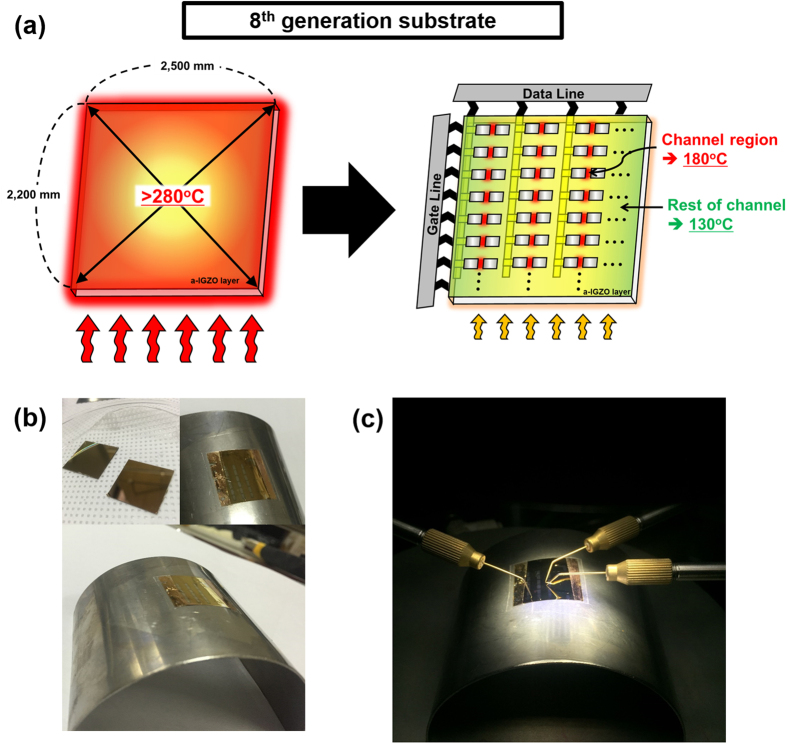
(**a**) Schematic illustration of (**a**) electrical activation comparison with conventional thermal activation. Photograph of (**b**) a-IGZO TFT on PI substrate, (**c**) electrical activation process of the a-IGZO TFT on PI substrate.

Conventional high temperature annealing heats all parts of the substrate, wasting a large amount of thermal energy in the active layer, which is etched away after activation. The selective electrical activation method enables efficient annealing focused on the predetermined areas defined in Fig. 1a. Simultaneous electrical and thermal treatment of the active layer induces localized Joule heating between the source and drain, resulting in no annealing of the peripheral areas outside of the channel region.

Through this novel technique, unwanted thermal damage of plastic substrates can be avoided when fabricating a-IGZO TFTs. Therefore, this new approach overcomes some limitations of the high temperature fabrication process required for conventional a-IGZO TFTs, and will enable the fabrication of large-area flexible display technology^28^.

A new technology for low temperature activation of the a-IGZO channel layer was developed, utilizing electrical energy in addition to thermal energy. The a-IGZO TFTs activated at low temperature (130 °C) exhibited superior electrical characteristics. Thermodynamic and electrical analysis showed that electrical activation may be promoted by two mechanisms. E_a_ is reduced with an applied electric field, and sufficient energy is provided by the sum of the electrical and thermal energies. The relationship between the self-heating effect and the local activation was discussed based on results from infrared microthermography. The annealing effect was focused in the channel region, and the temperature of the other areas was lower. As a result, this low temperature activation technique reduces 99% of the thermal budget by decreasing the anneal temperature and area. Electrical activation does not require the development of new equipment as existing facilities used for bias temperature stress testing can be used. This technique allows the use of inexpensive flexible substrates that have a low glass-transition temperature. Therefore, this research demonstrates the possibility of electric field-aided fabrication of a-IGZO TFTs at low temperatures, reducing fabrication costs and providing a great benefit to the flexible electronics community.

## Methods

### Fabrication of a-IGZO TFTs

**Figure 2:**
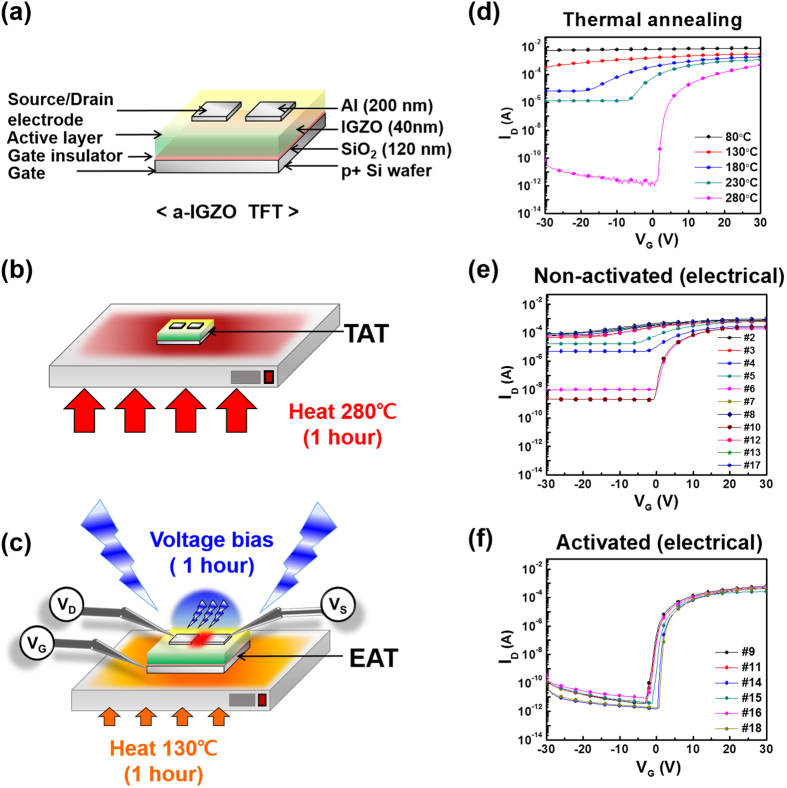
Schematic illustration of (**a**) a-IGZO TFT, (**b**) conventional activation via thermal annealing at 280 °C on hotplate for 1 hour, (**c**) electrical activation. Transfer characteristics of the a-IGZO TFTs, (**d**) annealed at 80, 130, 180, 230, and 280 °C, (**e**) non-activated (electrical) and (**f**) activated (electrical) samples.

**Figure 3:**
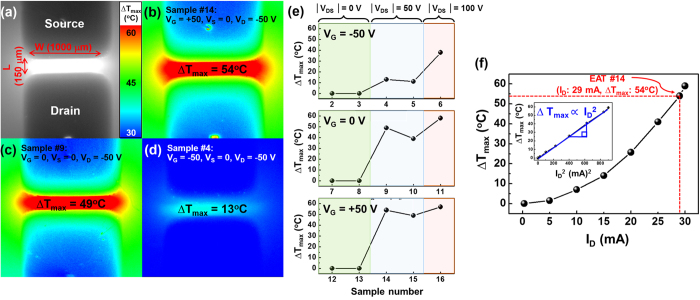
Infrared microthermography results. (**a**) Optical microscope image of a-IGZO TFT (sample number 14, V_G_ (+50) V_DS_ (−50)) Temperature distribution when V_S_ = 0, V_D_ = −50 V, and (**b**) V_G_ = +50 V (sample number 14, V_G_ (+50) V_DS_ (−50)) V_G_ = 0 V (sample number 9, V_G_ (0) V_DS_ (−50)) and (**d**) V_G_ = −50 V (sample number 4, V_G_ (−50) V_DS_ (−50)). (**e**) ∆T_max_ in the channel of a-IGZO TFTs under V_G_ = −50, 0, and +50 V conditions. (**f**) ∆T_max_ in the channel of a-IGZO TFT (sample number 14, V_G_ (+50) V_DS_ (−50)) as a function of I_D_. The inset graph shows ∆T_max_ versus the square of I_D_ under the same condition.

**Figure 4:**
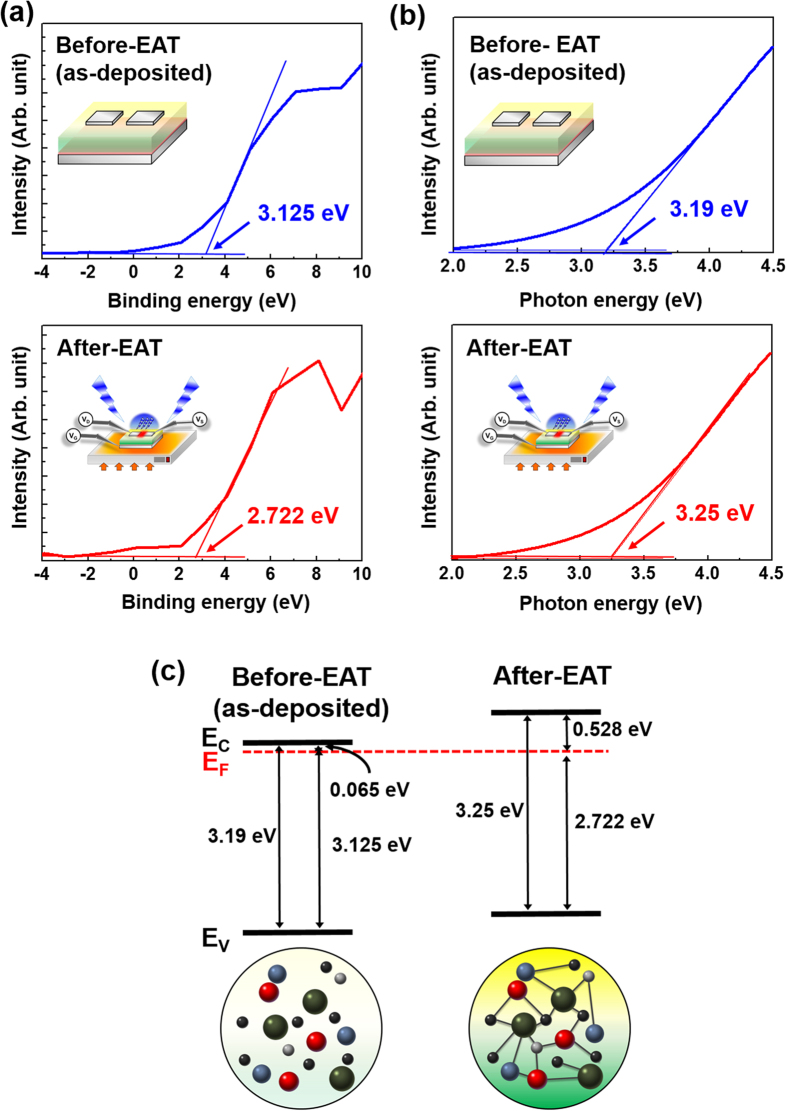
(**a**) XPS spectra near the valence band. (**b**) Imaginary part of SE spectra absorption coefficient. (**c**) Band alignment of before (as-deposited) and after electrical activation using XPS and SE data.

**Figure 5:**
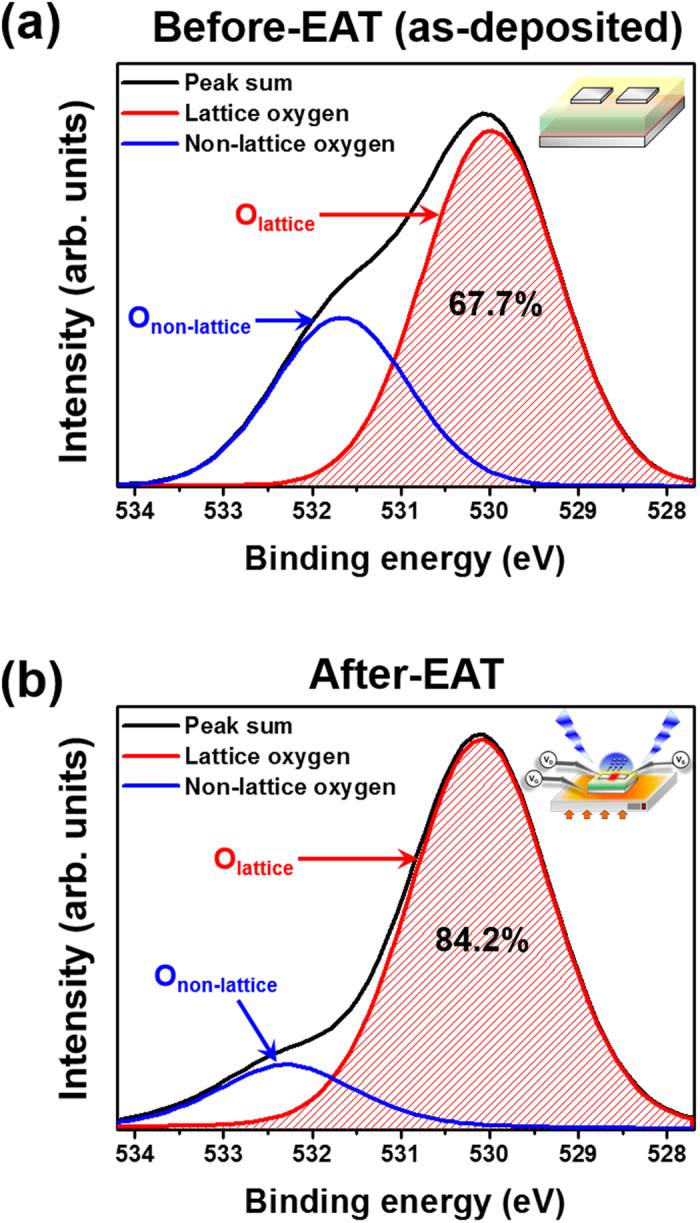
XPS results for the O 1s peak for (**a**) before (as-deposited) and (**b**) after electrical activation.

**Figure 6:**
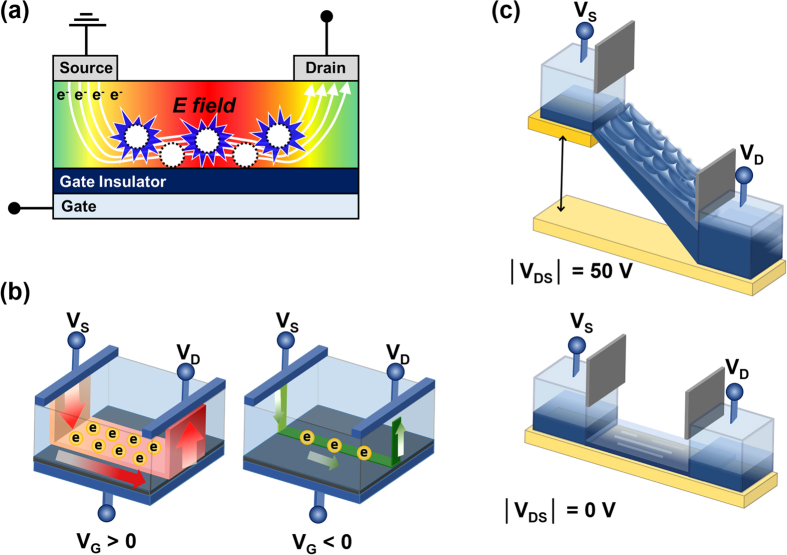
The schematic illustration of electrical activation mechanism related to Joule heating. (**a**) Joule heating from I_D_ during electrical activation, difference in (**b**) carriers between V_G_ > 0 and V_G_ < 0 and (**c**) I_D_ between |V_DS_| = 50 V and |V_DS_| = 0 V.

**Figure 7:**
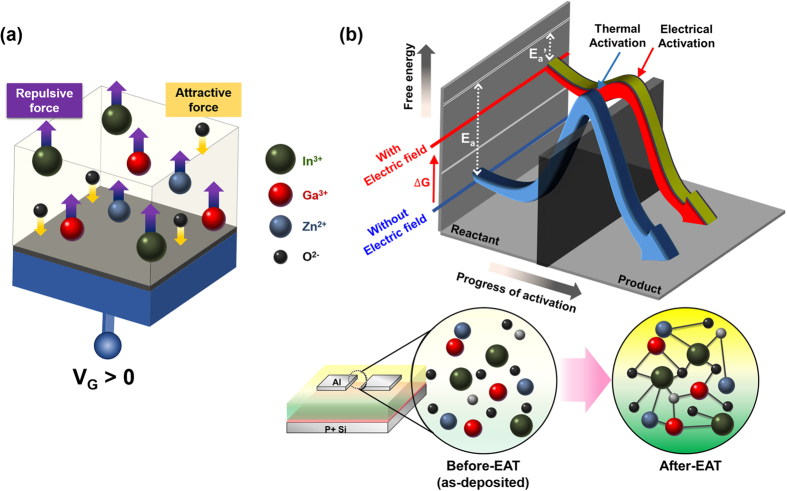
The schematic illustration of electrical activation mechanism related to electric field. (**a**) Coulomb forces between In^3+^, Ga^3+^, Zn^2+^, O^2−^ and positive V_G_. (**b**) Change in activation energy from E_a_ of TAT to E_a_’ of EAT.

a-IGZO TFTs were fabricated with an inverted staggered structure on a heavily doped p-type Si wafer with a 120-nm-thick layer of thermally grown SiO_2_. A 40-nm-thick a-IGZO active layer was deposited by radio frequency sputtering on the cleaned Si substrate using an IGZO target (In_2_O_3_:Ga_2_O_3_:ZnO = 1:1:1 mol%) at room temperature for 5 minutes. The 200-nm-thick aluminum source and drain electrodes were deposited by thermal evaporation, the channel region was defined with a width (W) of 1,000 μm, and a length (L) of 150 μm. Figure 2a shows a schematic of the a-IGZO TFTs. The conventional thermally activated TFTs (TATs, sample number 1) used as a reference were annealed at 280 °C for 1 hour in air, after the source and drain electrodes were patterned as shown in Fig. 2b. For clarity, the high-temperature annealing process described above will be referred to as ‘thermal activation’. A constant DC voltage was applied to the gate, drain, and source electrodes of the EATs for 1 hour. The samples were located inside a dark probe box to block light and fabricated at 130 °C, with the biases specified in Table S1. An illustration and photograph of the bias process are shown in Figs 1c and 2d. Figure 1b is a photograph of a-IGZO TFTs fabricated on the PI substrate. Figure 1c indicates that electrical activation on flexible substrates is feasible.

### Electrical, thermal, and chemical measurements

Electrical characteristics were measured in a dark box under ambient conditions using a semiconductor parameter analyzer (HP 4156C, Hewlett Packard, Palo Alto, CA, USA). TFT measurements were conducted by varying the gate voltage (V_GS_) from −30 V to 30 V, with a fixed drain voltage (V_DS_) of 10.1 V. High-resolution infrared microthermographic analysis was used to study localized heating of the samples, and the chemical composition of each film was analyzed with depth profile X-ray photoelectron spectroscopy (XPS). Spectroscopic ellipsometry (SE) was used for TFT band alignment.

## Additional Information

**How to cite this article**: Lee, H. *et al*. Electric Field-aided Selective Activation for Indium-Gallium-Zinc-Oxide Thin Film Transistors. *Sci. Rep.* **6**, 35044; doi: 10.1038/srep35044 (2016).

## Supplementary Material

Supplementary Information

## Figures and Tables

**Table 1:** Summary of the device electrical parameters, including μ_FET,_ on/off ratio, V_on_, SS, and N_max_ for a-IGZO TFTs (sample numbers 1~18).

#	Sample	Annealing temperature [°C]	V_G_ [V]	V_S_ [V]	V_D_ [V]	∆T_max_ [°C]	μ_FET_ [cm^2^ V^−1^ s^−1^]	I_on/off_ ratio	V_on_ [V]	SS [V decade^−1^]	N_max_ [cm^−3^]
1	TAT, reference	280	—	—	—	—	9.90	5.09 * 10^8^	−0.6	0.42	1.09 * 10^12^
2	V_G_ (−50) V_DS_ (0)_1	130	−50	0	0	0	—	1.83 * 10^1^	—	—	—
3	V_G_ (−50) V_DS_ (0)_2	130	−50	+50	+50	0	—	1.64 * 10^1^	—	—	—
4	V_G_ (−50) V_DS_ (−50)	130	−50	0	−50	13	8.08	5.79 * 10^1^	−1.6	6.30	1.88 * 10^13^
5	V_G_ (−50) V_DS_ (+50)	130	−50	0	+50	11	10.68	3.89 * 10^1^	−6.4	9.12	2.74 * 10^13^
6	V_G_ (−50) V_DS_ (+100)	130	−50	−50	+50	38	8.97	2.15 * 10^4^	−1.4	1.02	2.89 * 10^12^
7	V_G_ (0) V_DS_ (0)_1	130	0	−50	−50	0	—	1.07 * 10^1^	—	—	—
8	V_G_ (0) V_DS_ (0)_2	130	0	+50	+50	0	—	1.05 * 10^1^	—	—	—
9	V_G_ (0) V_DS_ (−50)	130	0	0	−50	49	13.75	1.81 * 10^8^	−3.4	0.59	1.59 * 10^12^
10	V_G_ (0) V_DS_ (+50)	130	0	0	+50	39	8.91	1.31 * 10^5^	−2.2	0.82	2.30 * 10^12^
11	V_G_ (0) V_DS_ (+100)	130	0	−50	+50	58	11.14	1.26 * 10^8^	−3.2	0.58	1.57 * 10^12^
12	V_G_ (+50) V_DS_ (0)_1	130	+50	−50	−50	0	—	1.09 * 10^1^	—	—	—
13	V_G_ (+50) V_DS_ (0)_2	130	+50	0	0	0	—	8.02	—	—	—
14	V_G_ (+50) V_DS_ (−50)	130	+50	0	−50	54	10.66	3.18 * 10^8^	−0.2	0.36	9.00 * 10^11^
15	V_G_ (+50) V_DS_ (+50)	130	+50	0	+50	49	11.23	7.19 * 10^7^	−1.4	0.49	1.30 * 10^12^
16	V_G_ (+50) V_DS_ (+100)	130	+50	−50	+50	57	11.87	6.23 * 10^7^	−2.4	0.55	1.49 * 10^12^
17	V_G_ (+50) V_DS_ (−50)_0.5 h	130	+50	0	−50	54	—	9.65	—	—	—
18	V_G_ (+50) V_DS_ (−50)_2 h	130	+50	0	−50	54	12.00	2.46 * 10^8^	0.2	0.36	8.92 * 10^11^
