# Supplementary material for: Electric Field-aided Selective Activation for Indium-Gallium-Zinc-Oxide Thin Film Transistors
Source: Sci Rep. 2016 Oct 11;6:35044. doi: 10.1038/srep35044 (PMC5057139; doi:10.1038/srep35044)

**Supplementary Information**

Electric Field-aided Selective Activation for Indium-Gallium-Zinc-Oxide Thin Film Transistors

Heesoo Lee^1^, Ki Soo Chang^2^, Young Jun Tak^1^, Tae Soo Jung^1^, Jeong Woo Park^1^, Won-Gi Kim^1^, Jusung Chung^1^, Chan Bae Jeong^2^, and Hyun Jae Kim^1^,*

**Table S1.** Process conditions for thermally and electrically activated a-IGZO thin films and increased temperatures caused by voltage bias effect.

| # | Annealing temperature  [^o^C] | V_G_  [V] | V_S_  [V] | V_D_  [V] | Treatment time  [hour] | │V_DS_ │  [V] | | V_GS_  [V] | V_GD_  [V] | ∆T_max_  [^o^C] |
| --- | --- | --- | --- | --- | --- | --- | --- | --- | --- | --- |
| 1 | 280 | - | - | - | 1 | - | - | | - | - |
| 2 | 130 | -50 | 0 | 0 | 1 | 0 | -50 | | -50 | 0 |
| 3 | 130 | -50 | +50 | +50 | 1 | 0 | -100 | | -100 | 0 |
| 4 | 130 | -50 | 0 | -50 | 1 | 50 | -50 | | 0 | 13 |
| 5 | 130 | -50 | 0 | +50 | 1 | 50 | -50 | | -100 | 11 |
| 6 | 130 | -50 | -50 | +50 | 1 | 100 | 0 | | -100 | 38 |
| 7 | 130 | 0 | -50 | -50 | 1 | 0 | +50 | | +50 | 0 |
| 8 | 130 | 0 | +50 | +50 | 1 | 0 | -50 | | -50 | 0 |
| 9 | 130 | 0 | 0 | -50 | 1 | 50 | 0 | | +50 | 49 |
| 10 | 130 | 0 | 0 | +50 | 1 | 50 | 0 | | -50 | 39 |
| 11 | 130 | 0 | -50 | +50 | 1 | 100 | +50 | | -50 | 58 |
| 12 | 130 | +50 | -50 | -50 | 1 | 0 | +100 | | +100 | 0 |
| 13 | 130 | +50 | 0 | 0 | 1 | 0 | +50 | | +50 | 0 |
| 14 | 130 | +50 | 0 | -50 | 1 | 50 | +50 | | +100 | 54 |
| 15 | 130 | +50 | 0 | +50 | 1 | 50 | +50 | | 0 | 49 |
| 16 | 130 | +50 | -50 | +50 | 1 | 100 | +100 | | 0 | 57 |
| 17 | 130 | +50 | 0 | -50 | 0.5 | 50 | +50 | | +100 | 54 |
| 18 | 130 | +50 | 0 | -50 | 2 | 50 | +50 | | +100 | 54 |

**Table S2.** Summary of the band alignment of before and after electrical activation.

|  | E_g_ | △(E_F_ - E_VB_) | △(E_CB_ - E_F_) |
| --- | --- | --- | --- |
| Pre-activation EAT | 3.19 eV | 3.125 eV | 0.065 eV |
| Post-activation EAT | 3.25 eV | 2.722 eV | 0.528 eV |

**Figure S1.** Variation of a) SS and b) µ_FET_ for a-IGZO TFTs (sample numbers 1, 4, 5, 6, 9, 10, 11, 14, 15, 16, and 18). The inset graph shows enlarged graph of a) SS vs sample numbers. 10 devices are measured for each sample. The error bars indicate the maximum, average, and minimum values of the 10 devices.


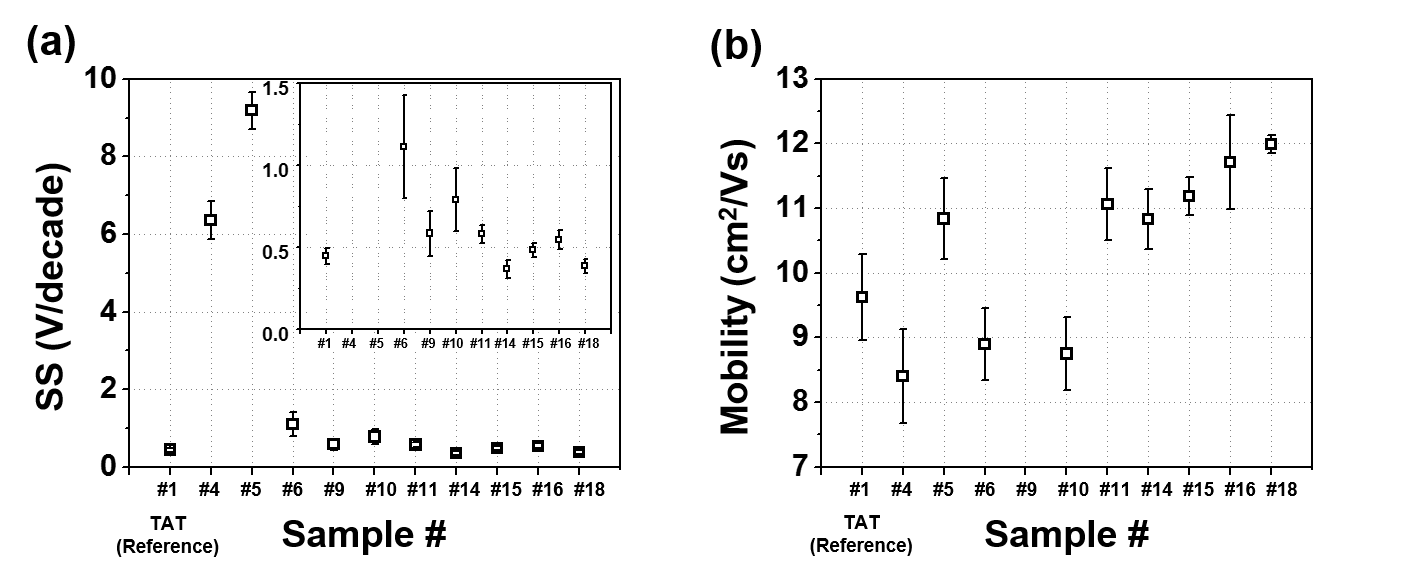


**Figure S2.** Transfer characteristics of the a-IGZO TFTs, activated (electrical) a) at RT, 80, 130, and 180 ^o^C, b) for 0.5, 1, and 2 hour, and c) in (V_G_, V_D_): (20, -20 V), (50, -50 V) and (100, -100 V).


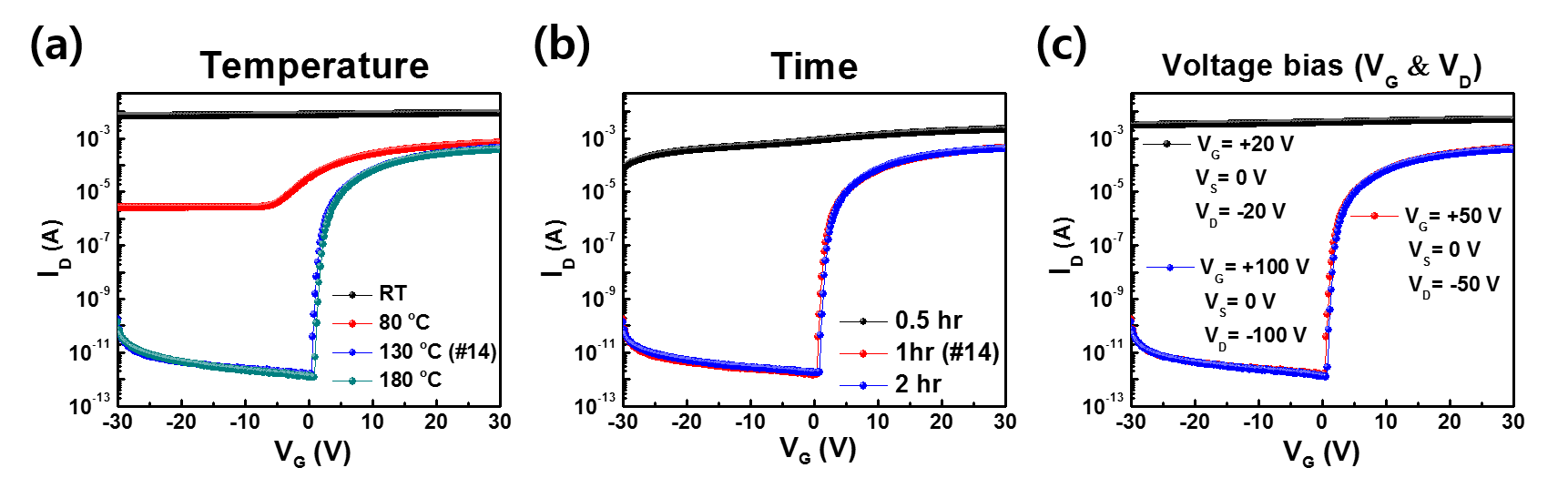

Supplement: Supplementary Information [file srep35044-s1.doc]
